# Comparative First-Principles Study of the Y_2_Ti_2_O_7_/Matrix Interface in ODS Alloys

**DOI:** 10.3390/ma17194822

**Published:** 2024-09-30

**Authors:** Yiren Wang, Dijun Long, Yong Jiang, Yongduo Sun

**Affiliations:** 1 Key Laboratory for Nonferrous Materials (MOE), School of Materials Science and Engineering, Central South University, Changsha 410083, China; yiren.wang@csu.edu.cn; 2 National Key Laboratory for Powder Metallurgy, Central South University, Changsha 410083, China; 3 Science and Technology on Reactor Fuel and Materials Laboratory, Nuclear Power Institute of China, Chengdu 610213, China

**Keywords:** ODS alloys, Y_2_Ti_2_O_7_, interface, first-principles

## Abstract

Oxide-dispersion-strengthened (ODS) alloys generally exhibit extraordinary service performance under severe conditions through the formation of ultrafine nano oxides. Y_2_Ti_2_O_7_ has been characterized as the major strengthening oxide in Fe-based ODS alloys. First-principles energetic analyses were performed to investigate the structural, elastic and interface properties of Y_2_Ti_2_O_7_ in either Fe-based or Ni-based ODS alloys. Y_2_Ti_2_O_7_ has comparable elastic constants to bcc-Fe and fcc-Ni and similar elastic deformation compatibility in Y_2_Ti_2_O_7_-strengthened Fe-based and Ni-based ODS alloys is therefore expected. The Ni/oxide interface has generally better thermostability than Fe/oxide across the whole range of the concerned oxygen chemical potential. Further interface bonding and adhesion calculations revealed that Y_2_Ti_2_O_7_ can enhance the bonding strength of Ni/Y_2_Ti_2_O_7_ through d-d orbital interaction between the interfacial YTi layer and Ni layer, while the interface bonding between the Fe layer and YTi layer is weakened compared to the metal matrix. First-principles calculations suggest that Y_2_Ti_2_O_7_ can be a candidate for strengthening nano-oxides in either Fe-based or Ni-based ODS alloys with well-behaved mechanical properties for fourth-generation fission reactors and further experimental validations are encouraged.

## 1. Introduction

Oxide-dispersion-strengthened (ODS) alloys with distinctive high-temperature mechanical properties and radiation damage resistance are considered promising candidate materials for use in fourth-generation nuclear reactors. High-density small-size (>10^22^~10^24^, <10 nm) oxide nanoparticles can precipitate through high-energy mechanical alloying (MA) of a pre-alloyed matrix with alloying solute powders and oxide powders [1,2,3]. The MA process dissolves the oxide constituents, which, later, can precipitate along with other alloying elements to form dispersive nanoclusters and nano-oxides during hot consolidation and subsequent heat treatments [4].

Among the several kinds of ODS alloys, Fe-based ODS alloys are one of the most well-studied. Various types of oxides have been introduced into the matrix materials as the source of O to form nano-sized dispersion oxide particles inside the material, such as SiO_2_, La_2_O_3_, MgO, ZrO_2_, Y_2_O_3_, etc. [5,6,7,8,9]. Y_2_O_3_ stands out due to its high thermal stability and low diffusion speed in an iron chromium matrix. The major strengthening oxides in Fe-based ODS alloys have been identified by TEM to be Y-Ti-O nanoparticles (mostly pyrochlore Y_2_Ti_2_O_7_), which are generally coherent or semi-coherent with the Fe matrix [10,11,12,13]. There have been numerous theoretical and experimental reports about ODS ferritic steels, and the extraordinary properties of the ODS steels are known to significantly depend on the oxide particles and oxide/matrix interfaces [14,15,16,17,18,19]. The nano-oxides can maintain phase stability under heavy irradiation while the oxide/metal interface can capture He atoms and, therefore, suppress the segregation of helium bubbles into grain boundaries. Commercial ODS ferritic steels like 14YWT-ODS with yield strengths of about 300 MPa measured at 800 °C show steady-state creep for about 38,555 h (4.4 years) [20]. However, the operation temperatures of Very-High-Temperature Reactors (VHTRs) or Gas-cooled Fast Reactors (GFRs) are expected to extend to 1273 K, much higher than other nuclear reactors [13,21]. Ni-based superalloys with ordered gamma-prime (γ′) phase (Ni3(Al, Ti)) as strengthening phases were initially considered as the candidate structural materials. However, the temperatures of VHTRs and GFRs exceed the γ′ coarsening or dissolution temperatures, which makes them more suitable for the intermediate temperature range rather than for temperatures significantly beyond 1000 °C [22,23,24]. Ni-based ODS alloys have thus been developed to be potential superior core materials in terms of their possible high-temperature creep resistance compared to ODS ferritic steels [25,26]. Al is the major alloy element in Ni-based ODS alloys. Unfortunately, a large Al addition can be detrimental to the mechanical performance of Ni-based ODS alloys with coarse Y-Al-O particles commonly observed due to the high affinity of Al to O [27,28,29]. These larger-size Y-Al-O nanoparticles generally have more irregular morphologies and sparser dispersion. The addition of other alloying elements to the metal matrix can be an effective way to overcome the problem by preferentially forming finer nano-oxides instead of Y-Al-O [30].

Y_2_Ti_2_O_7_ is characterized as the most effective strengthening oxide in Fe-based ODS alloys. The incorporation of Ti into the ferritic matrix can promote the formation of (Y-Ti-O) nanoclusters, and act as the precursors of the ultra-fine nano-oxides (mostly 1~10 nm), pyrochlore Y_2_Ti_2_O_7_ and orthorhombic Y_2_TiO_5_ [1,13,18,19,20,21]. Ti is another important alloying element used to form a gamma-prime phase in Ni superalloys and it presents comparable affinity to O. Therefore, Ti is introduced into Ni-based ODS alloys to obtain finer Y-Ti-O nano-oxides. High density (4 × 10^23^ m^−3^) and ultra-fine (3.2 nm) Y_2_Ti_2_O_7_ have been characterized in ODS-617 alloys, and the nanoprecipitates show good size stability over 1300 K [31]. The Y-Ti-O nano oxides can suppress the formation of coarse Y-Al-O particles and contribute to better high-temperature mechanical properties through composition design [32]. Therefore, Y_2_Ti_2_O_7_ is expected to play a similar role in Ni-based ODS alloys as in Fe-based systems, and the possibilities of Y_2_Ti_2_O_7_ as potential strengthening oxides in Ni-based ODS alloys need to be carefully examined.

Previous first-principles calculations have revealed that the bulk and interface properties of the strengthening nano-oxides determine the stability and radiation resistance of the ODS alloys, while the nucleation and growth of the nano-oxides are governed by the interfacial structures and the crystal correlations between the oxides and the matrix. Multiple microscopic characterizations have revealed the interface in Fe-based ODS alloys to be Y_2_Ti_2_O_7_ (001)/Fe(001) with different orientation relationships (ORs), including a cube-on-cube OR for thermally-coarsened Y_2_Ti_2_O_7_ as {100}<100>Fe//{100}<100>Y_2_Ti_2_O_7_ reported by Ribis et al. [33] and a Baker–Nutting OR of {100}<100>Fe//{100}<110> Y_2_Ti_2_O_7_ and cube-on-edge OR of {100}<100>Fe//{110}<100> Y_2_Ti_2_O_7_ reported by Ciston et al. and Dawson et al. [34,35] Previous first-principles thermodynamics investigations have shown that the non-stoichiometric interfaces (both Y/Ti- and O-rich) preferentially form with a much wider range of p_O2_, and the helium trapping at the Y/Ti-rich (ns-2Y2Ti) interface contributes to the remarkable irradiation tolerance [19,36]. Though fine Y_2_Ti_2_O_7_ particles are expected to contribute to a superior high-temperature mechanical strength in Ni-based ODS alloys, very few attempts have been reported to reveal the interface orientation. Mao et al. observed Y_2_Ti_2_O_7_ $\left(11\bar{1}\right)$/Ni(001) in the as-extruded Ni-based ODS 617 alloy through HRTEM, and yet, no detailed OR of Y_2_Ti_2_O_7_/Ni has been characterized [31]. Despite Y_2_Ti_2_O_7_ being generally known as a major strengthening oxide in Fe-based ODS alloys, the adhesion and bonding nature of the oxide/matrix interface remain unclear.

In this work, we investigate the potential of Y_2_Ti_2_O_7_ as the strengthening oxide in Ni-based ODS alloys through structural, elastic and interface calculations as well as controlled results of Y_2_Ti_2_O_7_ in Fe-based ODS alloys. The lattice, elastic and electronic nature of the oxide/matrix interface in ODS alloys is predicted using first-principles calculations to reveal the adhesion and bonding nature of the concerned oxide/matrix interfaces. With the development of nanomaterials, there might be potential for improvements by choosing different oxides in ODS alloys, such as Cu_2_O and α-Fe_2_O_3_ [37,38,39]. This study intends to provide a preliminary strategy for the evaluation of alternative oxides in ODS alloys.

## 2. Computational Details

All the DFT calculations were conducted using the Vienna ab initio Simulation Package (VASP) with the plane-wave basis sets and periodic boundary conditions [40]. The electron-core interaction was described by the Blöchl projector augmented wave method (PAW) within the frozen-core approximation [41]. The plane-wave basis sets were generated with valence configurations of Fe-3d^6^4s^2^, Ni-3d^8^4s^2^, Y-4s^2^4p^6^4d^1^5s^2^, Ti-5p^6^5d^2^6s^2^ and O-2s^2^2p^4^. All the ground-state configurations were optimized using a high-energy cut-off of 525 eV for the plane-wave basis sets until the total energy was minimized to 10^−5^ eV and the total force on each ion converged to within 0.02 eV/Å. All calculations are fully spin-polarized. The structural and electronic visualization was obtained by VESTA [42]. The local density approximation (LDA) [43], the Perdew–Wang-91 (PW91) [44] version and the Perdew–Burke–Ernzerhof (PBE) [45] version of the generalized gradient approximation (GGA) were employed in bulk properties calculations to describe the exchange-correlation functional.

## 3. Results and Discussions

### 3.1. Bulk Properties

**Figure 1:**
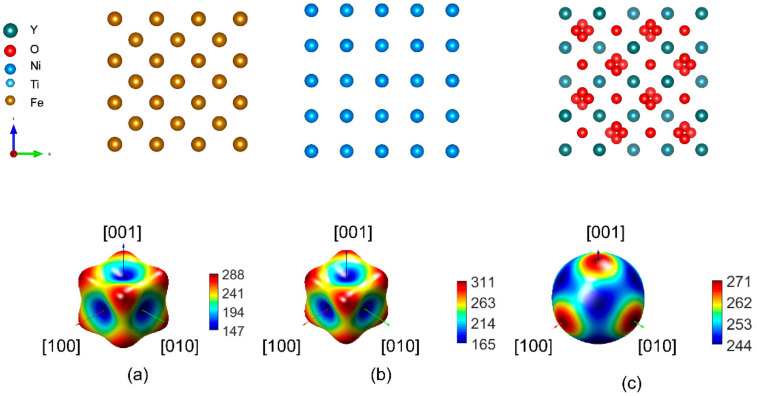
The atomic structure (**top**) and 3D plots of the mechanical quantities in the form of the calculated Young’s modulus (**bottom**) of the (**a**) bcc-Fe, (**b**) fcc-Ni, and (**c**) pyrochlore-Y_2_Ti_2_O_7_ using the GGA-PW91 functional.

For the Fe, Ni, and Y_2_Ti_2_O_7_ bulk calculations, 3 × 3 × 3, 4 × 4 × 4 and 1 × 1 × 1 supercell models, respectively, are adopted, as shown in Figure 1. Table 1 summarizes the corresponding calculated structural properties using a fitted third-order Birch–Murnaghan equation of states [46]. The linear elastic constants can be obtained from first principles with the derivatives of the stress as a function of strains parametrized by a set of distorted structures through a stress–strain methodology [47], and three simplified independent elastic constants (c_11_, c_12_ and c_4_) are obtained using the Voigt–Reuss–Hill approach [48,49]. The calculated lattice constants and bulk moduli using the GGA-PW91 method show good agreement with the available experiments and calculations in the literature and GGA-PW91 [41] was adopted for the exchange–correlation functionals in the subsequent calculations. Y_2_Ti_2_O_7_ is regarded as an ideal strengthening oxide in ODS steel since it presents superior mechanical properties and its bulk modulus is very close to that of bcc-Fe, therefore, a good coordinated deformation compatibility can be expected with the ferritic matrix. Interestingly, our bulk calculations suggest that the predicted c_ij_ values of pyrochlore-Y_2_Ti_2_O_7_ are favorably comparable to those of bcc-Fe and fcc-Ni, suggesting that pyrochlore Y_2_Ti_2_O_7_ may have similar elastic deformation compatibilities with the Ni matrix as with Fe matrix. The ratio of bulk to shear moduli B/G was proposed by Pugh to predict the brittle or ductile behaviors of materials [50]. If B/G > 1.75, a ductile behavior can be predicted for the crystalline alloy systems; otherwise, the material shows a brittle behavior. Moreover, according to Pettifor’s criterion, a material with intrinsic ductility has a positive Cauchy pressure, C′ = C12 − C44 > 0; whereas, a material with a negative Cauchy pressure (C′ < 0) is intrinsically brittle [51,52]. Both Pugh and Pettifor criteria describe ductile behaviors and the calculated B/G ratio of Y_2_Ti_2_O_7_ is rather close to that of the metal matrix as well, indicating similar ductile deformation behaviors can be observed in Fe, Ni and Y_2_Ti_2_O_7_. Please note, this prediction describes the pristine materials only, the actual mechanic behaviors of ODS alloys may be influenced by the service environment.

Based on the calculated elastic constants, the elastic anisotropy of the concerned bulk structures can be evaluated by calculating the Zener anisotropy ratio Z = 2C44/(C_11_–C_12_) (where Z = 1 means isotropic) [59]. The anisotropy indices indicate both Fe and Ni bulk materials are anisotropic in nature. As shown by the graphical presentation of Young’s modulus shown in Figure 1, Y_2_Ti_2_O_7_ is almost elastically isotropic but slightly stiffer along the [100] direction than along [111] (the maximum Young’s modulus is along [100]) while more obvious elastic anisotropic behavior can be observed in bcc-Fe and fcc-Ni. According to our calculations, the elastic modulus of Y_2_Ti_2_O_7_ including bulk modulus, Young′s modulus and shear modulus, are similar to those of bcc-Fe and fcc-Ni, which indicates that the dispersed nano-oxide can deform accordingly with the matrix and superior ductility is expected during the elastic deformation stage.

### 3.2. Interface Structural Properties

ODS alloy performance relies significantly on the strengthening oxides and their interface with the matrix. Investigations of the interface bonding and strength are necessary to evaluate the potential for Y_2_Ti_2_O_7_ as strengthening oxides in ODS alloys. Detailed structural information is required to construct the metal/oxide interface including the interface orientation relation (OR), interfacial strain, atomic coordination, etc.

The cube-on-cube OR of Fe(100)/Y_2_Ti_2_O_7_ (100) is the most commonly observed OR among the abundant characterizations, while the only observed interface orientation of Y_2_Ti_2_O_7_ in Ni-based ODS alloys is (100)_Ni_||$\left(11\bar{1}\right)$_Y2Ti2O7_. Previous interface evaluations of Y_2_Ti_2_O_7_ in Fe-based ODS alloys have suggested that the cube-on-cube ORs are thermodynamically Y/Ti-rich, therefore, an interface sandwich model with 262 atoms is built as Fe(100) [001]//Y_2_Ti_2_O_7_ (100) [001]. There are nine layers of Fe atoms and a total of seven blocks of the Y/Ti-rich Y_2_Ti_2_O_7_. The Ni(100) [011]//Y_2_Ti_2_O_7_ $\left(11\bar{1}\right)$ $\left[2\bar{1}0\right]$ with the minimum interfacial strain is constructed as well, with 231 atoms including nine layers of Ni atoms and eight blocks of Y/Ti-rich Y_2_Ti_2_O_7_. Please note that the atomic coordination of the interface models is chosen based on supercell energy optimizations.

**Figure 2:**
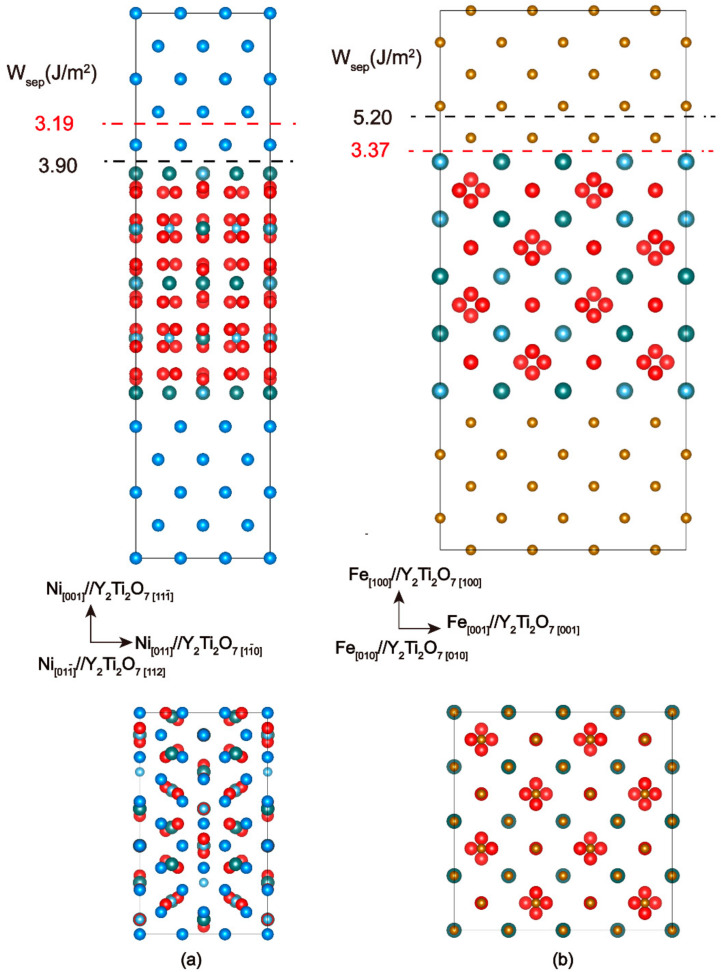
Schematic interface models for the constructed metal/oxide interfaces from the front and top views. (**a**) Ni(001) [011]//Y_2_Ti_2_O_7_ $\left(11\bar{1}\right)$ $\left[1\bar{1}0\right]$, and (**b**) Fe(100) [001]//Y_2_Ti_2_O_7_ (100) [001] interfaces. The top images show the front views of the interface models with the OR directions and the bottom images are the corresponding top views.

The resulting interface supercells after full relaxation are shown in Figure 2, including one Y_2_Ti_2_O_7_ $\left(11\bar{1}\right)$ (1 × 1) cell matching with one Ni (100) (5 × 3) cell (Figure 2a), and one Fe (100) (4 × 4) cell matching with one Y_2_Ti_2_O_7_ (100) (1 × 1) cell (Figure 2b). The commensuration strains for the three concerned interface models are summarized in Table 2. The interface structures and properties are generally believed to be related to the coherency and both matrix and oxide are stretched or compressed to match the interface structure since good deformation compatibilities are predicted by the bulk calculations. Better coherency is found in the Ni/Y_2_Ti_2_O_7_ model than the Fe/Y_2_Ti_2_O_7_ interface with a smaller interface mismatch.

Interface energies (γ) are calculated to evaluate the thermodynamic stabilities of the systems using the following equation,

(1) $$\gamma =\frac{1}{2A}(E_{interface}-n_{M}\mu_{M}-n_{O}\mu_{O}-n_{Ti}\mu_{Ti}-n_{Y}\mu_{Y})$$

where $E_{interface}$ represents the total energy of the metal/oxide interface, $\mu_{M},$ and $\mu_{O}$, $\mu_{Ti}$ and $\mu_{Y}$ are the chemical potentials of a single metal, O, Ti and Y atom, respectively. $n_{M}$, $n_{O}$, $n_{Ti}$, and $n_{Y}$ are the numbers of the corresponding atoms in the constructed interface models. A is the interface area and the numerical 1/2 indicates there are two interface sections in each sandwich supercell model.

The chemical potentials of matrix Fe or Ni atoms are taken as the energy per atom in its bulk phase while other elements shall meet the following relation,

(2) $$E_{Y2Ti2O7}=7\mu_{O}+2\mu_{Ti}+2\mu_{Y}$$

here, $E_{Y2Ti2O7}$ is the energy of the Y_2_Ti_2_O_7_ unit cell.

The atomic ratio of Y and Ti atoms remains 1:1 in our interface model, that is $n_{Y}=n_{Ti}$. Therefore, Equation (1) can be rewritten as

(3) $$\gamma =\frac{1}{2A}[E_{interface}-n_{M}\mu_{M}-\frac{n_{Y}}{2}E_{Y2Ti2O7}+(\frac{{7n}_{Y}}{2}-n_{O}{)\mu }_{O}]$$

The upper limit of the oxygen chemical potential is taken as the corresponding energy of O in the metal oxide FeO or NiO since the ODS-alloy system should avoid oxidation of the metal matrix; while the lower limit for oxygen is considered to oxidize the metallic titanium into Y_2_Ti_2_O_7_ from Y_2_O_3_. Therefore, the chemical potential range for oxygen is taken as,

(4) $$(E_{Y2Ti2O7}-E_{Y2O3}-2\mu_{Ti})/4<\mu_{O}<E_{MO}-\mu_{M}$$

**Figure 3:**
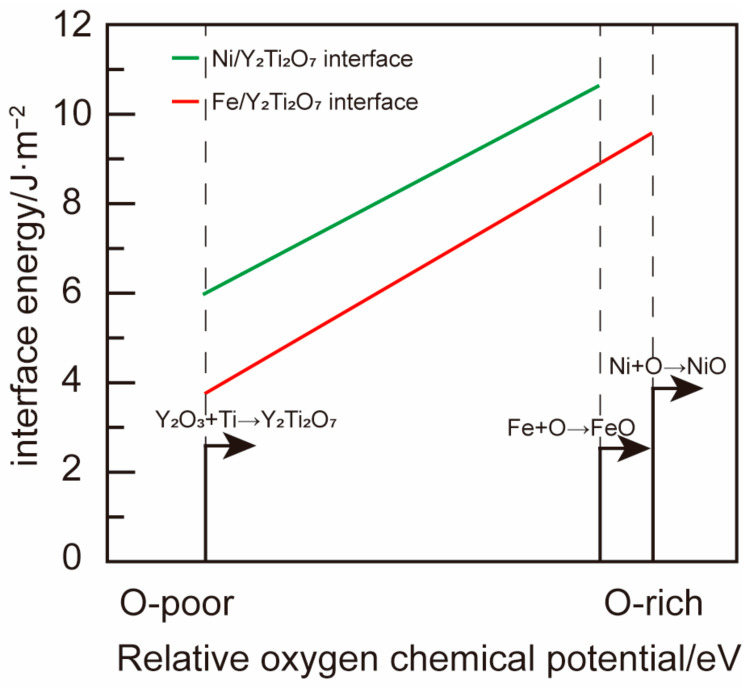
Calculated interface energies of the concerned metal/oxide interfaces in Fe-based and Ni-based ODS alloys as functions of oxygen chemical potential.

The calculated interface formation energies of the metal/oxide interfaces are shown in Figure 3 where the Ni/oxide interface generally has a lower γ than Fe/oxide across the whole range of the concerned oxygen chemical potentials. Considering the observed coherent interface structure and good thermostability of the Fe/Y_2_Ti_2_O_7_ interface, the Ni/Y_2_Ti_2_O_7_ interface is believed to show comparable thermostability with potentially enhanced mechanical performance induced by the interface structures.

### 3.3. Interface Adhesion Properties

The plastic deformation ability of an alloy system can be significantly influenced by the interface region in the system. The interfacial adhesion strength in terms of the work of separation (W_sep_) is, therefore, evaluated by calculating the cleavage energy required to rigidly separate the interface ensemble into two halves with an infinite distance,

$$W_{sep}=-\frac{1}{2A}(E_{interface}-E_{A}-E_{B})$$

here, $E_{interface}$ represents the total energy of the metal/oxide interface, and $E_{A}$ and $E_{B}$ are the energies of the counterparts that separate from the interface supercell with a one-step static calculation.

**Figure 4:**
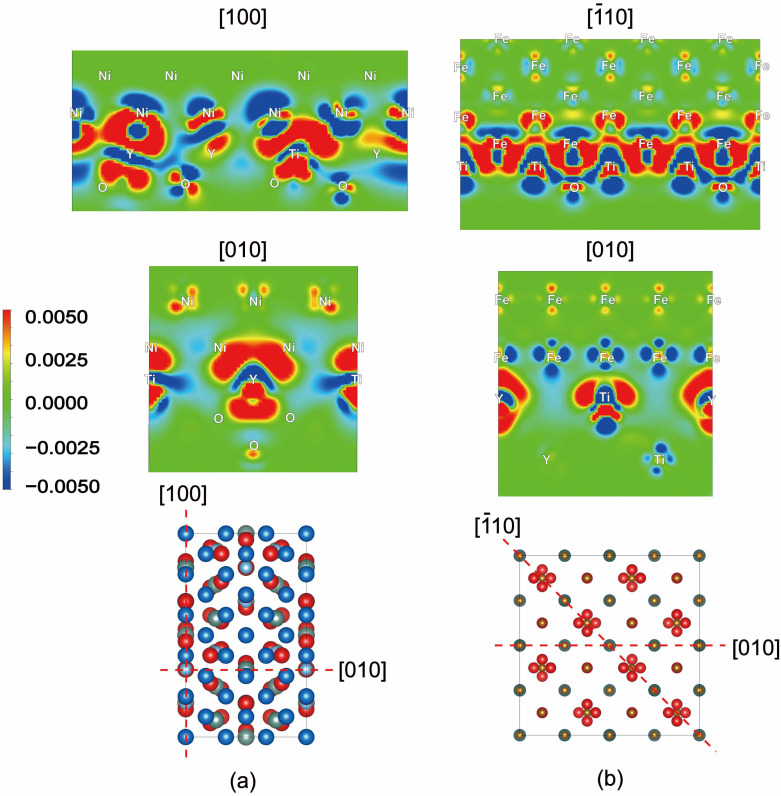
Charge density difference contours (in units of e/Å^3^) of (**a**) Ni/Y_2_Ti_2_O_7_ interface and (**b**) Fe/Y_2_Ti_2_O_7_ interface from different directions as denoted in the corresponding atomic models.

Strong adhesion is evidently found in both matrix/Y_2_Ti_2_O_7_ interfaces as shown in Figure 4. For the considered Ni/Y_2_Ti_2_O_7_ interface, the weakest interlayer binding does not take place right at the oxide/matrix interface, but inside the sub-interface layer close to the Ni metal lattice. A crack initiated at the Ni/Y2Ti2O7 interface is more likely to grow towards the Ni matrix and then result in the failure of the material. Despite the experimentally reported strengthening effects of Y_2_Ti_2_O_7_ in an Fe matrix, the formation of Y_2_Ti_2_O_7_ in an Fe matrix results in a decrease in the interface adhesion compared to the sub-interface metallic bonding, indicating that the crack propagation direction tends to be along the interface. W_sep_ was calculated as high as 3.19 J/m^2^ for the Ni/Y_2_Ti_2_O_7_ interface, and 3.37 J/m^2^ for the Fe/Y_2_Ti_2_O_7_ interface. The interfacial bonding strength of Ni/Y_2_Ti_2_O_7_ is higher than that of the Ni atom layer, suggesting stronger strengthening adhesion of the Ni matrix can be achieved by Y_2_Ti_2_O_7_. Despite the Fe/Y_2_Ti_2_O_7_ interface layer being weakened compared to the Fe sublayer, it displays a comparable fracture strength as most metals with W_sep_ over 3.0 J/m^2^. The decrease in the interface layer can be attributed to the interfacial strain induced by the structures. Moreover, the work of separation of the Y_2_Ti_2_O_7_ sublayer for either metal/oxide interface is calculated to be rather high (more than 7.0 J/m^2^), mainly as a result of the covalent bonding nature of the oxide. Combined with the calculation of elasticity properties, the preliminary deformation behaviors of the Y_2_Ti_2_O_7_-strengthened Fe- or Ni-based alloys can be predicted. At the initial stage of deformation, the ODS alloy is subjected to elastic deformation. The introduced elastically isotropic Y_2_Ti_2_O_7_ can deform continuously and accordingly with the metal matrix. As the amount of deformation increases, plastic deformation begins to occur. The cleavage fracture shall initiate from the interfacial atomic layer of the Ni/Y_2_Ti_2_O_7_ interface and the sub-atomic layer of the Fe/Y_2_Ti_2_O_7_ interface.

The bonding and electronic properties of oxide/matrix interfaces are further investigated by calculating the charge density difference between the interface ensemble and the superposition densities of the two component halves, as shown in Figure 4. There are substantial charges distributed between the metal atoms and oxide, and the charge density is obviously larger than that of the metallic matrix. A large charge redistribution region is observed between the oxide and metal atom interface region due to a d-d orbital interaction between the interfacial YTi layer and Ni layer. Y and Ti have at least two unpaired d electrons, so they can form covalent bonds directly with the p*_z_* orbitals of nearby oxygen atoms. The charge gain zone of the Ni/oxide interface is in the first atomic layer in the metal matrix, while the accumulation of charges also occurs at Fe atoms above the interface region, which is consistent with the Wsep result that the Fe atomic layer requires more energy to cleave. A more covalent nature of the Ni/Y_2_Ti_2_O_7_ interface than the Fe/Y_2_Ti_2_O_7_ interface is observed, resulting in the enhancement of the interfacial bonding strength compared to the metallic layers.

**Figure 5:**
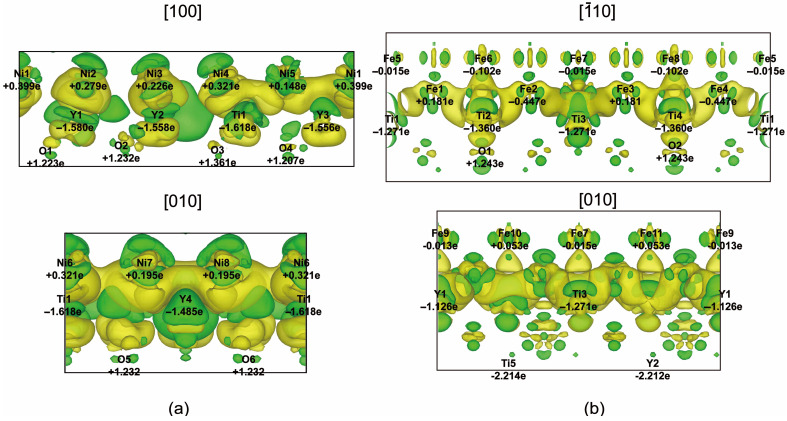
Bader charge density contours (in units of 0.005 e/Å^3^) of the concerned (**a**) Ni/Y_2_Ti_2_O_7_ interface and (**b**) Fe/Y_2_Ti_2_O_7_ interface from different directions as denoted in the differential charge densities.

As shown in Figure 5, Bader charge analysis further confirms the net transfer of valence charge from interfacial YTi-layer atoms towards the sub-interfacial O layers, which can be attributed to the larger electronegativity of O than Y and Ti. According to the oxide/Fe interface model, the interfacial Ti atom loses an average of ~1.315 e/atom and Y loses ~1.157 e/atom, while the sublayer O atoms gain ~1.243 e/atom. Although some of the Fe atoms may lose electrons, interfacial Fe atoms generally gain an average of ~0.090 e/atom. Matrix Fe atoms are more inert than matrix Ni atoms since the charge transfer in sublayer Ni atoms is less than 0.023 e/atom, while interfacial Ni atoms at the oxide/Ni interface are more active by gaining about ~0.234 e/atom on average. At the same time, certain amounts of charge dissipation occur above interfacial Fe atoms, and this accords well with the obvious charge redistribution region in Figure 4. Due to the difference in electronegativity, interfacial Y and Ti lose an average of ~1.570 e/atom and ~1.618 e/atom, respectively, while O gains ~1.248 e/atom. Evidently, the interfacial atoms are linked by newly formed bonds that are rather delocalized across the interfaces, and a mixed covalent and ionic bond character can be observed. The charge accumulation of metal atoms in the Ni/Y_2_Ti_2_O_7_ interface is obviously larger than that at the Fe/Y_2_Ti_2_O_7_ interface, which is caused by the large electronegativity difference between Ni and Y/Ti atoms. In addition, the charge distribution around the Y and Ti atoms is slightly stronger as well. The resulting remarkably enhanced interface bonding in the Ni/Y_2_Ti_2_O_7_ interface is consistent with its maximum work of separation calculated above. The more active charge transfer between the oxide/Ni interface than that in the oxide/Fe interface results in strong interface adhesion and increased covalent bonding.

## 4. Conclusions

Oxide-dispersed-strengthened alloys have shown great potential as structural materials in nuclear fission reactors, and the properties of the alloys rely on the type of the oxides and their interface properties with the matrix. In this study, structural, elastic and interface properties of Y_2_Ti_2_O_7_ in either Fe-based or Ni-based ODS alloys are discussed based on first-principles calculations. Comparable elastic constants for Y_2_Ti_2_O_7_ with those of bcc-Fe and fcc-Ni are obtained, and a similar elastic deformation compatibility with the nickel superalloy matrix as with the ferritic matrix is, therefore, expected. An investigation of the thermostability of interface energies suggests that the oxide/metal interface can be more easily formed in a Ni matrix than in an Fe matrix across the whole range of the concerned oxygen chemical potentials. Further interface bonding and adhesion calculations revealed that Y_2_Ti_2_O_7_ can enhance the bonding strength of Ni/Y_2_Ti_2_O_7_ through a d-d orbital interaction between the interfacial YTi layer and Ni layer, while the interface bonding between the Fe and YTi layers is weakened compared to the metal matrix. Y_2_Ti_2_O_7_ is known as the major and most effective strengthening oxide in Fe-based ODS alloys with well-behaved interfacial mechanical performance. Therefore, the primary theoretical feasibility of Y_2_Ti_2_O_7_-strengthened Ni-based ODS alloy is proposed by first-principles calculations while further experimental validations of the creep strength and radiation-damage resistance are strongly encouraged.

## Figures and Tables

**Table 1:** Calculated structural and mechanical properties of bulk fcc-Ni, bcc-Fe and pyrochlore-Y_2_Ti_2_O_7_ in comparison to available results in the literature.

Material		GGA- PW91	GGA- PBE	LDA	Other Calc.	Expt.
pyrochlore-Y_2_Ti_2_O_7_	*a/*Å	10.18	10.19	10.00	10.17, 10.11 [15]	10.09 [53]
	*B*_0_ */GPa*	183	184	214	183, 193 [15]	
	*c*_11_	334	331	368	325 [15]	
	*c*_12_	119	117	140	111 [15]	
	*c*_44_	95	93	110	76 [15]	
	B/GPa	191	188	216	182 [15]	170, 190, 192 [54]
	G/GPa	100	98	111	87 [15]	101, 103, 104 [54]
	E/GPa	255	251	285	226 [15]	253, 62, 265 [54]
	B/G	1.91	1.92	1.94	2.09 [15]	0.25, 0.27 [54]
	v	0.28	0.28	0.28	0.29 [15]	1.70~1.90 [54]
	Z	0.88	0.87	0.96	0.71 [15]	
Fcc-Ni	*a/*Å	3.52	3.52	3.43	3.52, 3.53 [55]	3.52 [56]
	*B*_0_ */GPa*	197	198	242	189 [55]	
	*c*_11_	271	279	301	276 [55]	252 [57]
	*c*_12_	149	155	271	160 [55]	154 [57]
	*c*_44_	127	130	143	126 [55]	122 [57]
	*B/GPa*	190	197	214	199 [55]	187 [57]
	*G/GPa*	95	97	105	92 [55]	85 [57]
	*E/GPa*	243	249	270	240 [55]	220 [57]
	B/G	2.00	2.03	2.04	2.15 [55]	2.21 [57]
	v	0.29	0.29	0.29	0.30 [55]	0.30 [57]
	Z	2.08	2.10	2.18	2.17 [55]	
bcc-Fe	*a/*Å	2.84	2.84	2.75	2.83 [15]	2.87 [56]
	*B*_0_ */GPa*	175	177	256	175 [15]	
	*c*_11_	243	241	257.26	236 [15]	243 [58]
	*c*_12_	135	134	153.07	132 [15]	138 [58]
	*c*_44_	118	118	132.29	105 [15]	122 [58]
	B/GPa	173	173	189	166 [15]	173 [58]
	G/GPa	86	87	93	84 [15]	94 [58]
	E/GPa	221	225	240	205 [15]	238 [58]
	B/G	1.98	1.97	2.03	2.1 [15]	0.26 [58]
	v	0.28	0.28	0.29	0.29 [15]	1.84 [58]
	Z	2.19	2.20	2.54	2.02	

**Table 2:** The commensuration strains induced in Ni/Y_2_Ti_2_O_7_ and Fe/Y_2_Ti_2_O_7_ interfaces.

Ni(001)/Y_2_Ti_2_O_7_ $\left(11\bar{1}\right)$	Fe(100)/Y_2_Ti_2_O_7_ (100)
U_Ni_ = +0.27% V_Ni_ = +1.27%	U_Fe_ = +3.35% V_Fe_ = +3.35%
U_227_ = −0.04% V_227_ = −2.59%	U_227_ = −7.43% V_227_ = −7.43%

## Data Availability

The raw data supporting the conclusions of this article will be made available by the authors on request.
